# The contribution of malaria control interventions on spatio-temporal changes of parasitaemia risk in Uganda during 2009–2014

**DOI:** 10.1186/s13071-017-2393-0

**Published:** 2017-09-30

**Authors:** Julius Ssempiira, Betty Nambuusi, John Kissa, Bosco Agaba, Fredrick Makumbi, Simon Kasasa, Penelope Vounatsou

**Affiliations:** 1 Swiss Tropical and Public Health Institute, Basel, Switzerland; 2 University of Basel, Basel, Switzerland; 3 Ministry of Health, Kampala, Uganda; 4 Makerere University School of Public Health, Kampala, Uganda

**Keywords:** Malaria, Malaria indicator survey, Spatio-temporal, Parasitaemia, ITNs, IRS, ACTs, Spatially varying, Bayesian kriging, Malaria interventions

## Abstract

**Background:**

In Uganda, malaria vector control interventions and case management with Artemisinin Combination Therapies (ACTs) have been scaled up over the last few years as a result of increased funding. Data on parasitaemia prevalence among children less than 5 years old and coverage of interventions was collected during the first two Malaria Indicator Surveys (MIS) conducted in 2009 and 2014, respectively. In this study, we quantify the effects of control interventions on parasitaemia risk changes between the two MIS in a spatio-temporal analysis.

**Methods:**

Bayesian geostatistical and temporal models were fitted on the MIS data of 2009 and 2014. The models took into account geographical misalignment in the locations of the two surveys and adjusted for climatic changes and socio-economic differentials. Parasitaemia risk was predicted over a 2 × 2 km^2^ grid and the number of infected children less than 5 years old was estimated. Geostatistical variable selection was applied to identify the most important ITN coverage indicators. A spatially varying coefficient model was used to estimate intervention effects at sub-national level.

**Results:**

The coverage of Insecticide Treated Nets (ITNs) and ACTs more than doubled at country and sub-national levels during the period 2009–2014. The coverage of Indoor Residual Spraying (IRS) remained static at all levels. ITNs, IRS, and ACTs were associated with a reduction in parasitaemia odds of 19% (95% BCI: 18–29%), 78% (95% BCI: 67–84%), and 34% (95% BCI: 28–66%), respectively. Intervention effects varied with region. Higher socio-economic status and living in urban areas were associated with parasitaemia odds reduction of 46% (95% BCI: 0.51–0.57) and 57% (95% BCI: 0.40–0.53), respectively. The probability of parasitaemia risk decline in the country was 85% and varied from 70% in the North-East region to 100% in Kampala region. The estimated number of children infected with malaria declined from 2,480,373 in 2009 to 825,636 in 2014.

**Conclusions:**

Interventions have had a strong effect on the decline of parasitaemia risk in Uganda during 2009–2014, albeit with varying magnitude in the regions. This success should be sustained by optimizing ITN coverage to achieve universal coverage.

**Electronic supplementary material:**

The online version of this article (doi: 10.1186/s13071-017-2393-0) contains supplementary material, which is available to authorized users.

## Background

Although malaria is still a leading global health problem, its burden has been on a decline in recent years [1]. This decline which started in the early 1990s prior to the global campaign of scaling up of control interventions in mid-2000s continued through the post-scale-up period [2]. The downward trend of malaria burden in the pre-intervention period notwithstanding, sufficient evidence from randomized trials and field settings indicate that malaria decline during the post-scale-up period has been unprecedented [2–5]. For instance in sub-Saharan Africa (SSA) parasitaemia prevalence declined from 17% in 2010 to 13% in 2015 [1]. Also, during the period 2000–2015, declines in global malaria incidence and deaths of up to 37 and 60%, respectively were reported [3, 6]. These declines were mainly attributed to the impact of Insecticide Treated Nets (ITNs) and malaria case management with Artemisinin Combination Therapies (ACTs).

In spite of these higher declines in malaria at global level, slower declines were reported in the 15 most high burden countries, the majority of which are situated in SSA [1]. This region bears the heaviest burden and accounts for an estimated 90% of all malaria deaths mainly among children less than 5 years old. Uganda is ranked fourth among these high malaria burden countries and has some of the highest malaria transmission rates in the world [7]. Since 2006, Roll Back Malaria (RBM) has funded malaria control and prevention activities in the country and periodically supports the conducting of Malaria Indicator Surveys (MIS) [8]. The MIS are standardized nationally representative surveys that collect high quality data for estimating the prevalence of parasitaemia risk in children less than 5 years old and track the progress of interventions coverage. To date, two MIS have been conducted in Uganda; MIS 2009 and MIS 2014–15 [9, 10]. Findings from the first MIS revealed a high parasitaemia risk in most regions. Malaria was hyperendemic (prevalence 50–75%) in three regions, mesoendemic (prevalence 10–50%) in six, and only hypoendemic (prevalence < 10%) in one region [10]. Results of the second MIS showed tremendous improvement in the coverage of ITNs and ACTs intervention at all levels and a reduction of parasitaemia risk of 50%. Additionally, parasitaemia risk in the majority of regions had declined to mesoendemic and hypoendemic proportions [9]. The true effect of each intervention on parasitaemia reduction is not known at national and sub-national level, and yet a new framework has been adopted by the Ministry of Health (MoH) to speed up malaria control efforts. In this framework known as Uganda Malaria Reduction Strategic Plan (UMRSP) 2014–2020, ambitious targets have been set to reduce malaria mortality to near zero, morbidity to 30 cases per 1000 population, and parasite prevalence to less than 7% [8]. To achieve these targets and ensure efficient use of scarce resources and effective programming and implementation, it is vital to understand the effect that each intervention has had on parasitaemia risk decline.

Declines in malaria parasitaemia risk, morbidity and mortality have been achieved in other malaria endemic countries following scaling up of control interventions. Bhatt et al. [3] reported a reduction of 50% in *Plasmodium falciparum* prevalence and 40% in incidence of clinical disease in endemic African countries between 2000 and 2015. Similarly, the number of malaria cases and deaths decreased by more than 50% in southern African countries after introducing interventions during 2000–2008 [11]. In the Kilifi district of Kenya, parasitaemia prevalence declined from 35 to 1% after a mass distribution of ITNs and ACTs [11]. Also, Giardina et al. [12] demonstrated that ITNs and IRS were significantly associated with parasitaemia risk reduction in Rwanda, Tanzania, Senegal, Angola, Liberia and Mozambique.

Our study aims to estimate spatio-temporal trends of parasitaemia risk changes among children less than 5 years old in Uganda during 2009–2014, and to determine the effect of interventions on parasitaemia risk decline at national and subnational levels. We analyzed MIS data using Bayesian spatio-temporal geostatistical models. The results from this study provide insight on the effectiveness of interventions and can be used by MoH and Malaria Control Program (MCP) to evaluate interventions and optimize resources for achievement of objectives of UMRSP 2014–2020.

## Methods

### Country profile

Uganda is located in the great lakes region in East Africa neighboring Kenya, Tanzania, Rwanda, Democratic Republic of Congo, and South Sudan. It has a population of 37.1 million, all of which are at risk of malaria. Malaria is the leading cause of morbidity and mortality in the country, accounting for 3,631,939 (4,400,000–12,000,000) cases and 5921 (5300–17,000) deaths in 2015 [13]. The most dominant malaria parasite is *Plasmodium falciparum*, and the major transmission vectors are *Anopheles gambiae* and *Anopheles funestus*. In recent times, vector resistance to both pyrethroid and carbamates has been reported.

### Data sources

Parasitological and interventions data were obtained from the MIS data of 2009 and 2014–2015. The two surveys were conducted at the peak of a high malaria transmission season towards the end of the long rainy season (December 2009 and December 2014–January 2015, respectively). The MIS are nationally representative surveys which employ a two-stage stratified cluster design. The clusters also known as census enumeration areas are selected at first stage with probability proportional-to-size sampling, and households are selected at second stage using systematic sampling. The surveys are designed to provide information on key malaria control indicators, such as the proportion of households having at least one ITN, the proportion of children under 5 years of age who slept under an ITN the previous night. Also, the survey is designed to produce key indicator estimates for urban and rural strata separately, as well as for the 10 regions/domains that constitute the country. The regions are: Kampala, Central 1, Central 2, East-Central, Mid-North, Mid-Western, North-East, South-Western and West Nile. At the first stage of sampling, 170 and 210 clusters were selected in 2009 and 2014, respectively. At the second stage, 28 households were selected from each cluster in both surveys resulting in a total of 4000 and 5880 households selected in the first and second survey, respectively [9, 10].

Coverage of ITNs was defined in terms of ownership and use indicators that were generated from data captured on the survey tools using standard definitions [14]. The following ITN ownership indicators were defined; proportion of households with at least one ITN, proportion of households with one ITN for every two people, and proportion of population with access to an ITN within their household. The ITN use indicators were: proportion of children less than 5 years of age who slept under an ITN, proportion of population that slept under an ITN, and proportion of ITNs used the night preceding the survey. IRS coverage was defined as the proportion of households that were sprayed during the last 12 and 6 months in the MIS 2009 and MIS 2014–2015, respectively. The wealth index derived from household possessions was used as a socioeconomic proxy. A case management indicator was defined as the proportion of fever episodes in children of less than 5 years old during the last 2 weeks preceding the survey which were treated with any Artemisinin Combination Therapies (ACTs). In addition, information on the location of the cluster (i.e. rural/urban) was obtained from survey data and from the Global Rural-Urban Mapping Project (GRUMP) database [15]. The GRUMP database provides gridded data at 1 km^2^ spatial resolution.

Malaria transmission depends on the environment which affects the disease distribution, seasonality, and transmission intensity. Environmental/climatic factors were extracted from Remote Sensing (RS) sources. Weekly day and night Land Surface Temperature (LST), bi-weekly Normalized Difference Vegetation index (NDVI) and land cover data were obtained from Moderate Resolution Imaging Spectroradiometer (MODIS) at 1 km^2^ spatial resolution. Dekadal rainfall data at 8 × 8 km^2^ resolution were extracted from the US Early Warning and Environmental Monitoring System (EWES). Altitude was obtained from the shuttle radar topographic mission using the digital elevation model. Also, distances from cluster centroids to major water bodies were estimated using ESRI’s ArcGIS 10.2.1 for Desktop. The high spatial resolution population data was downloaded from WorldPop [16]. Data from remote sensing sources was acquired for the 12 month period preceding the survey and the average (cumulative value for rainfall) was calculated and extracted for each cluster. The one-year period was considered long enough to capture the actual climatic conditions that affected malaria transmission throughout the year of the survey.

### Statistical analysis

Bayesian geostatistical models were developed to predict parasitaemia risk at the two survey time points using environmental/climatic factors as predictors. Bayesian kriging was applied to obtain parasitaemia risk estimates over a 2 × 2 km^2^ resolution grid. Predictions were used to determine the probability of parasitaemia risk reduction between the two surveys.

The number of children infected with malaria in the two surveys was estimated by combining high spatial resolution population data obtained from WorldPop (www.worldpop.org) with the predicted pixel-level malaria prevalence estimates. The number of children less than 5 years old was estimated by multiplying population counts by a factor of 17.7%, the proportion of population under 5 years of age [17]. Regional estimates of the number of infected children were computed by aggregating pixel-level estimates at regional level. The number of infected children per pixel was obtained by multiplying pixel-wise spatially explicit prevalence estimates with high spatial resolution population estimates of number of children less than 5 years old. In both surveys, the population-adjusted prevalence was estimated by summing up estimates of the number of infected children per pixel divided by the total estimated number of children less than 5 years old.

The effects of interventions were estimated by modeling the change of parasitaemia risk between the two surveys on the logit scale as a function of the effect of intervention coverage at the second survey adjusted for socioeconomic status, cluster location, and the difference in environmental/climatic factors. Geographical misalignment of the locations between the two surveys was carried out by predicting parasitaemia risk of the first survey at the second survey locations. The prediction uncertainty was incorporated by fitting an error term in the model. A spatially varying coefficients model was used to estimate intervention effects at regional level and to account for potential interactions of interventions with endemicity level.

A spike and slab geostatistical Bayesian variable selection procedure was applied to select the most important ITN and environmental predictors that explain maximum variation in the change in parasitaemia risk between 2009 and 2014 [18]. Variables with the highest inclusion probability in the model were selected.

Descriptive analyses were carried out in STATA (StataCorp. 2015. Stata Statistical Software: Release 14. College Station, TX: StataCorp LP). Geostatistical modeling was implemented in OpenBUGS version 3.2.3 (Imperial College and Medical Research Council, London, UK). Since implementing Bayesian kriging in OpenBUGS is very slow especially for large grids, we implemented it in R statistical software using posterior estimates of the model parameters obtained from OpenBUGS. Maps were produced in ESRI’s ArcGIS 10.2.1 (http://www.esri.com/en-us/home).

Parameter estimates were summarized by their posterior medians and their corresponding 95% Bayesian Credible Intervals (BCI). The effect of a predictor was considered to be statistically important if its 95% BCI did not include zero.

Detailed explanations of the fitted statistical models are presented in Additional files 1 and 2.

## Results

**Fig. 1:**
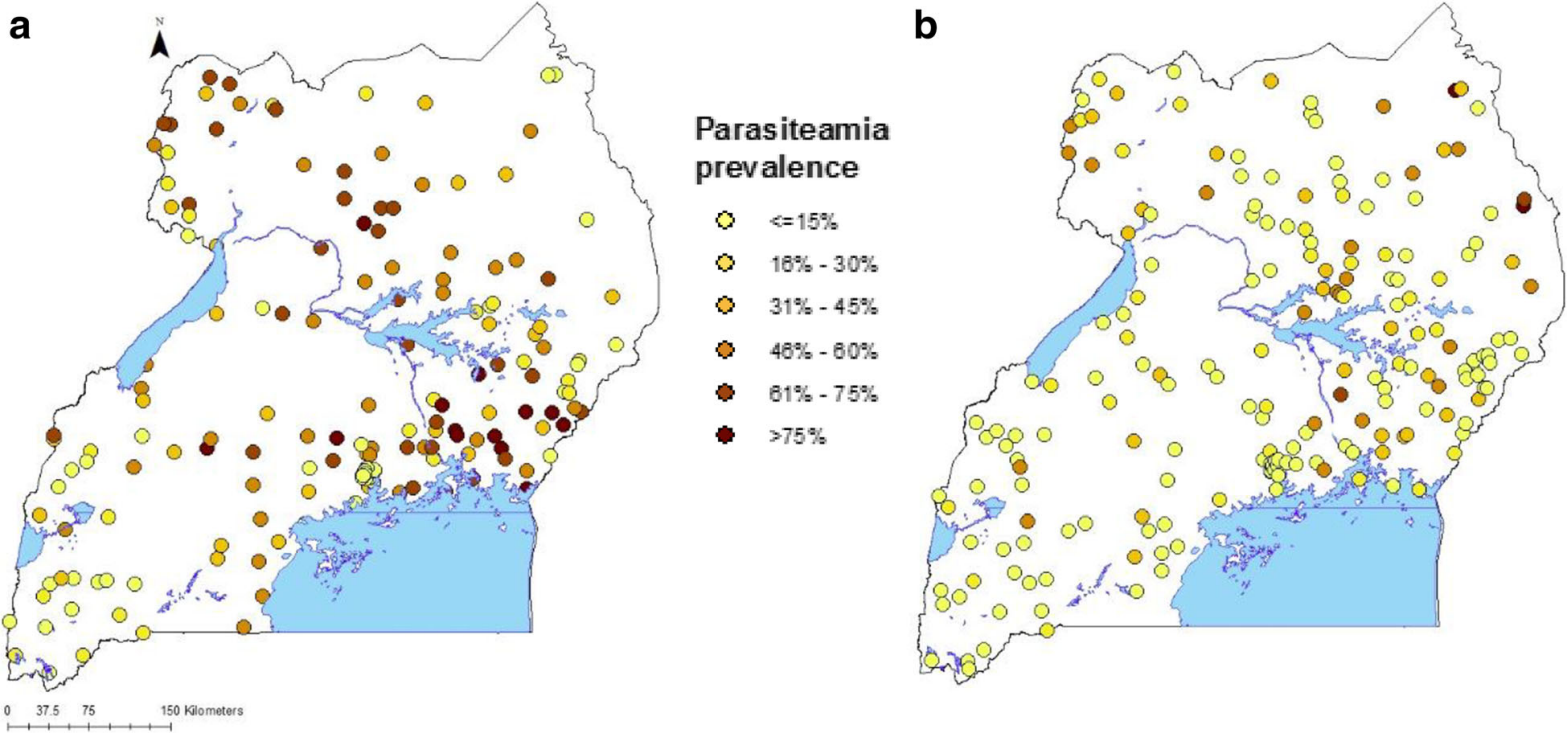
Observed malaria prevalence and survey locations of MIS 2009 (**a**) and MIS 2014–2015 (**b**)

A summary of the survey data is given in Tables 1 and 2, and maps of survey locations are presented in Fig. 1. A higher number of clusters, households, and children were tested in the second survey (Table 1).

**Table 1:** Survey information and malaria intervention coverage indicators in 2009 and 2014

Indicator	MIS 2009	MIS 2014–2015
Number of clusters	170	210
Number of households	4421	5345
Number of children tested	3972	4939
Interventions	% (95%CI)	% (95% CI)
Parasitaemia prevalence	42.4 (37.7–47.0)	19.0 (16.3–21.8)
Proportion of households with at least one ITN	46.7 (42.7–50.6)	90.2 (88.7–91.7)
Proportion of households with at least one ITN for every two people	16.4 (14.2–18.5)	62.3 (60.1–64.5)
Proportion of population with access to an ITN in their household	32.2 (29.3–35.1)	80.6 (78.9–82.4)
Proportion of the population that slept under an ITN the previous night	26.3 (23.5–29.2)	70.8 (68.9–72.8)
Proportion of children less than 5 years old who slept under an ITN the previous night	32.9 (29.0–36.9)	74.5 (72.2–76.9)
Proportion of existing ITNs used the previous night	26.1 (23.3–28.9)	70.4 (68.5–72.4)
Proportion of households sprayed in the last 6 months	5.5 (3.0–7.9)	5.2 (3.4–6.9)
Proportion of households with at least one ITN and/or sprayed by IRS in the last 12 months	49.2 (45.3–53.1)	90.5 (89.0–92.0)
Proportion of fever episodes treated with ACT	23.3 (19.9–26.7)	66.8 (63.2–70.5)

*Abbreviations*: *MIS* Malaria Indicator Survey, *TNs* Insecticide Treated Nets, *ACTs* Artemisinin Combination Therapies, *IRS* Indoor Residual Spraying

**Table 2:** Coverage of malaria intervention coverage indicators by region in 2009 and 2014

Indicator	Central 1		Central 2		Kampala		East-Central		Mid-Eastern		North-East		Mid-North		West Nile		Mid-Western		South-Western	
	2009	2014	2009	2014	2009	2014	2009	2014	2009	2014	2009	2014	2009	2014	2009	2014	2009	2014	2009	2014
Parasitaemia prevalence	39.0	10.4	51.0	23.6	4.9	0.4	56.2	36.4	37.4	13.5	39.7	27.2	62.1	19.5	45.6	27.5	42.7	17.5	11.8	4.1
Proportion of households with at least one ITN	35.3	80.8	23.5	81.6	49.1	86.3	33.5	82.1	59.5	94.6	76.6	97.0	63.7	94.3	52.4	96.3	33.9	93.6	33.7	96.9
Proportion of households with at least one ITN for every two people	14.6	56.7	9.3	53.4	32.4	66.5	7.8	46.7	17.0	61.7	33.1	60.6	20.1	66.7	12.8	72.1	12.1	64.0	14.7	76.6
Proportion of population with access to an ITN	25.4	71.8	16.4	70.8	42.4	79.2	21.6	68.7	37.1	83.7	57.0	84.2	43.7	85.8	33.0	88.8	23.0	83.7	30.0	91.1
Proportion of the population that slept under an ITN	19.1	60.1	10.3	58.6	36.9	73.0	18.7	62.8	31.4	76.3	54.3	85.5	32.1	77.6	33.1	77.7	17.0	78.6	22.6	67.0
Proportion of children less than 5 years old who slept under an ITN	21.5	67.6	11.3	65.3	42.5	73.9	19.3	69.7	41.4	78.8	65.1	87.0	41.7	79.0	37.2	76.8	20.4	82.3	33.1	64.4
Proportion of existing ITNs used the previous night	19.1	59.6	10.3	58.5	36.7	72.7	18.7	62.6	31.1	75.9	52.5	84.3	32.0	77.1	32.9	77.0	16.8	78.5	22.6	66.6
Proportion of households sprayed	0.2	1.0	4.6	0.4	5.5	1.3	0.4	0.0	0.6	0.4	4.2	0.1	31.6	44.6	0.0	1.2	0.2	0.3	1.8	0.0
Proportion of households with at least one ITN and/or sprayed by IRS in the last 12 months	35.3	80.8	26.3	81.9	52.3	86.3	33.8	82.1	59.6	94.6	77.1	97.0	77.8	97.2	52.4	96.3	34.1	93.6	44.7	96.9
Proportion of fever episodes treated with any artemisin combination therapy	17.4	55.2	18.0	71.7	22.5	51.5	13.4	71.1	16.6	68.0	25.1	73.3	40.8	69.2	27.7	67.0	19.4	61.1	10.0	53.3

*Abbreviations*: *ITNs* Insecticide Treated Nets, *ACTs* Artemisinin Combination Therapies, *IRS* Indoor Residual Spraying

Results show that at country level parasitaemia prevalence declined from 42.4% in 2009 to 19.0% in 2014, a decline of 50%. At regional level, the highest malaria reduction was observed in the regions of Kampala (91.8%), Central 1 (74.0%) and Mid-North (68.6%), and the lowest in the North-East region (30.2%) and East-Central region (35.2%).

Generally interventions coverage increased at country and regional levels (Additional file 3). At country level, ITN ownership (the proportion of households with at least one ITN and the proportion of households with at least one ITN for every two people) increased by four-fold. Among regions, the biggest increase in ITN ownership was reported in East-Central (six-fold), while the smallest was observed in Mid-North (two-fold). More so, the proportion of children less than 5 years old that slept under an ITN increased by more than two times at country level. The improvement in this indicator coverage was highest in Central 2 region (5.8 times) and lowest in North-East region (1.3 times).

Overall, the proportion of fever episodes treated with ACTs increased by three times. The highest increase was achieved in South-Western, East-Central and West Nile regions where coverage increased by more than five times. The least gain in ACTs coverage was observed in Mid-North region where it increased by almost two times. The national IRS coverage remained static at 5% except in the Mid-North region where an increase of 41% was achieved.

### Spatio-temporal trends of parasitaemia risk during 2009–2014

The effects of the most important environmental factors identified through geostatistical variable selection are shown in Table 3. Results indicate that more environmental factors were related to parasitaemia risk in 2009 compared to 2014. Also, spatial correlation was stronger in 2009.

**Table 3:** Posterior estimates of the effect of environmental factors on parasitaemia risk in 2009 and 2014

Predictor	MIS 2009	MIS 2014–2015
	OR (95% BCI)	OR (95% BCI)
Day LST^b^		
< 27.84 / < 31.4	1	1
27.84–30.18 / 31.4–33.8	1.68 (1.44–2.14)^a^	2.75 (2.03–3.64)^a^
> = 30.19 / > = 33.8	1.41 (1.28–1.76)^a^	2.19 (1.79–3.39)^a^
Night LST	1.55 (1.39–1.67)^a^	1.44 (1.19–1.60)^a^
Area type		
Rural *vs* urban	7.80 (4.88–11.09)^a^	3.70 (2.56–4.88)^a^
NDVI	1.25 (1.10–1.51)^a^	
Rainfall^b^		
< 17.11 / < 17.14	1	
17.11–18.49 / 17.14–18.79	1.13 (0.93–1.23)	
> = 18.50 / > = 18.79	1.39 (1.12–1.49)^a^	
Altitude^b^		
< 1098	1	
1098–1201	0.89 (0.81–0.95)^a^	
> = 1202	0.43 (0.38–0.47)^a^	
Land cover		
Others		1
Crops		1.19 (1.13–1.43)^a^
Spatial parameters		
Spatial variance	1.12 (0.99–1.20)	0.54 (0.49–0.59)
Range (km)	43.3 (12.2–57.8)	43.8 (36.3–48.2)

*Abbreviations*: *MIS* Malaria Indicator Survey, *LST* Land Surface Temperature, *NDVI* Normalized Difference Vegetation Index

^a^Statistically important effect

^b^Cut-offs before and after the slash (/) are for 2009 and 2014 respectively

**Fig. 2:**
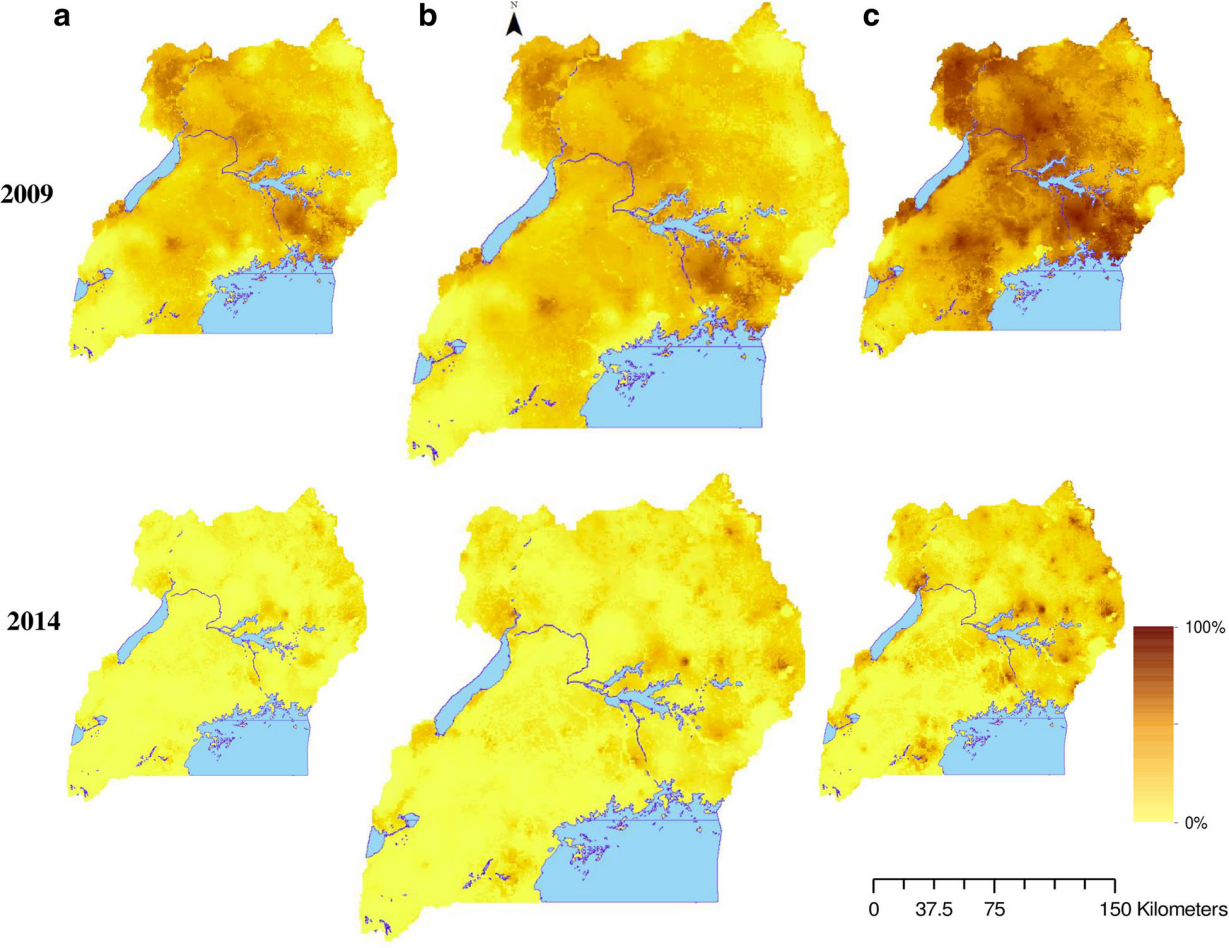
Predicted parasitaemia risk in 2009 and 2014. 2.5th percentile posterior predictive distribution (**a**), median posterior predictive distribution (**b**), 97.5th percentile posterior predictive distribution (**c**)

**Fig. 3:**
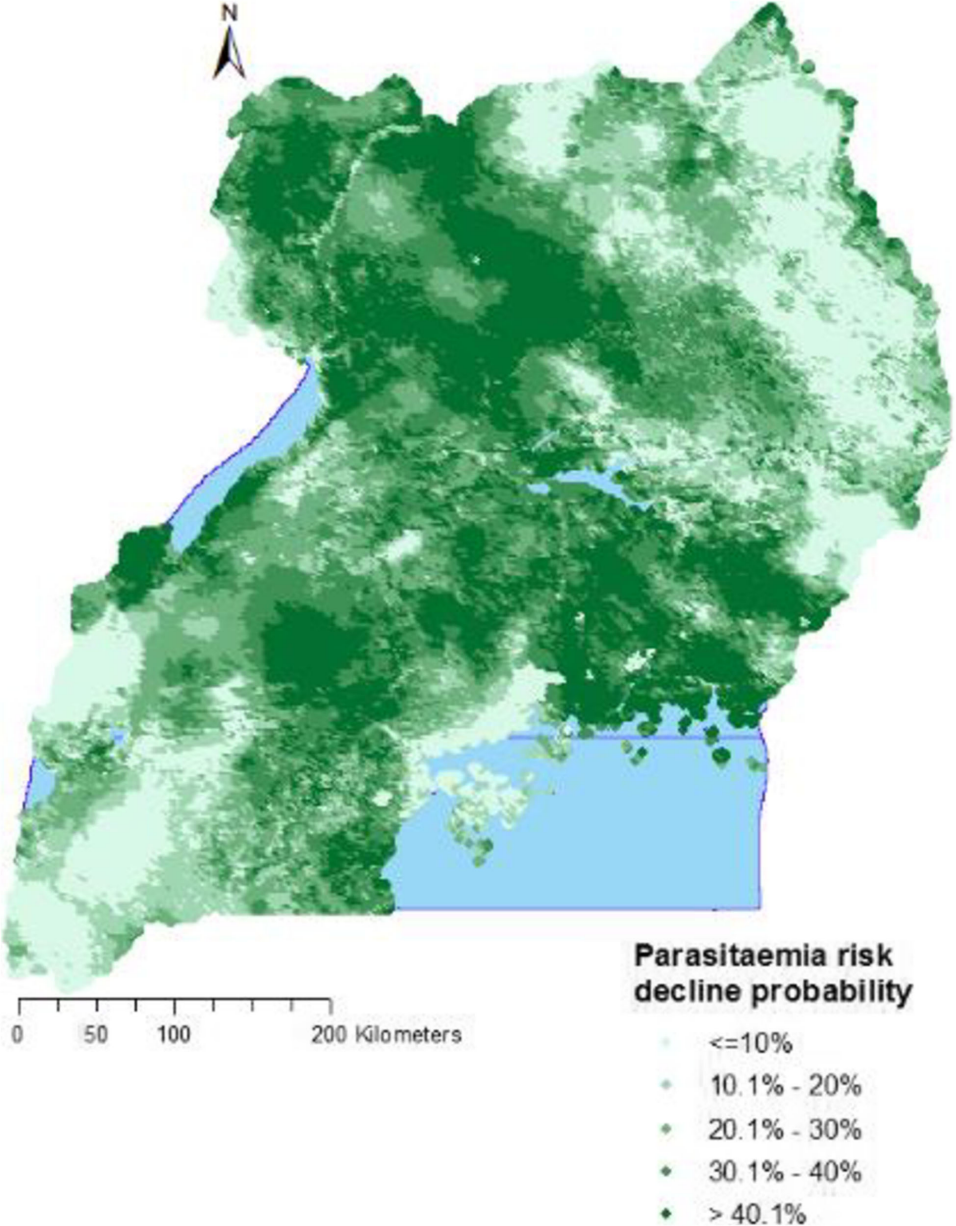
Probability of parasitaemia risk decline from 2009 to 2014

Figure 2 depicts the predicted parasitaemia risk in 2009 and 2014 over a 2 × 2 km^2^ resolution grid based on the 2.5th, median, and the 97.5th percentile posterior predictive distributions. Estimates suggest a high parasitaemia risk in 2009 where in some areas the predicted prevalence was over 80%. In 2014, parasitaemia risk was much lower in most parts of the country except in some areas of the East-Central, North-East and West Nile regions where the burden still remained high. The probability of parasitaemia decline in the country was 85%. The highest decline in malaria occurred in the regions of Central 2 and Kampala while the least was estimated in the North-East region (Fig. 3).

Overall, the number of infected children reduced from over 2,480,000 to less than 830,000 between 2009 and 2014 (Table 4). This translates into a reduction of over 66%. Reduction in the estimated number of infected children was achieved in all regions. The biggest reduction occurred in Kampala (86%), Central 1 (75%), Central 2 (74%), Mid-Eastern (71%) and Mid-North region (70%), whereas the least happened in North-East (44%). In both surveys, the highest and lowest numbers of infected children were estimated in the East-Central and Kampala regions, respectively. Overall, a reduction in population adjusted prevalence of over 26% was achieved. The highest reduction (39.4%) was observed in the East-Central region while the least one (5.0%) was registered in Kampala.

**Table 4:** Estimated number of infected children and population adjusted prevalence in 2009 and 2014

Region	No. of infected children in 2009	No. of infected children in 2014	Percentage reduction in no. of infected children	Population adjusted prevalence in 2009	Population adjusted prevalence in 2014	Population adjusted prevalence difference
			(%)	% (95% BCI)	% (95% BCI)	(%)
North-East	212,159	119,871	43.5	37.6 (37.4–37.8)	23.3 (23.1–23.4)	14.3
West Nile	276,237	106,377	61.5	56.8 (56.4–57.2)	25.8 (25.5–26.0)	31.0
Mid-North	332,162	98,846	70.2	52.4 (52.2–52.5)	20.0 (19.8–20.2)	32.4
Mid-Western	269,487	77,027	71.4	39.6 (39.3–39.9)	12.9 (12.7–13.1)	26.7
Mid-Eastern	274,376	79,734	70.9	46.3 (45.6–47.1)	16.8 (16.4–17.2)	29.5
East-Central	375,575	138,191	63.2	64.7 (64.3–65.1)	25.3 (24.8–25.8)	39.4
Central 2	338,097	87,562	74.1	50.1 (49.8–50.3)	14.4 (14.2–14.6)	35.7
Central 1	232,426	58,314	74.9	38.2 (37.8–38.6)	10.6 (10.4–10.8)	27.6
South-Western	148,799	56,819	61.7	22.2 (22.0–22.5)	8.8 (8.6–9.1)	13.4
Kampala	21,060	2895	86.3	5.9 (5.2–6.5)	0.9 (0.8–1.1)	5.0
Overall	2,480,373	825,636	66.7	44.0 (43.9–44.2)	17.7 (17.6–17.7)	26.3

**Fig. 4:**
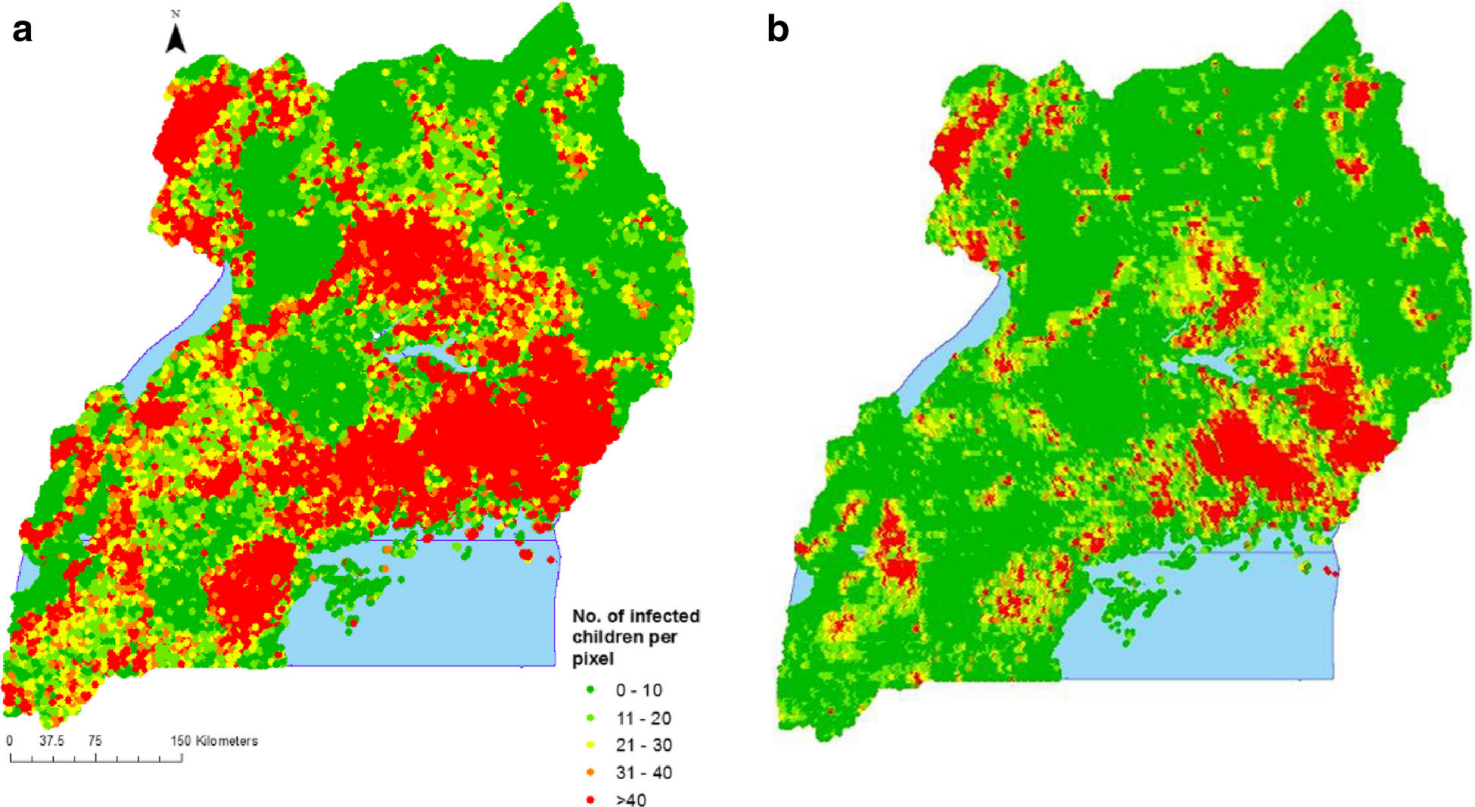
Distribution of estimated number of infected children per pixel in 2009 (**a**) and 2014 (**b**)

Figure 4 further shows that the number of infected children in 2014 shrank considerably compared to 2009 in all regions except in the East-Central region. The map also depicts a strong statistically important reduction in concentration of infected children in Mid North region in 2014.

Results from geostatistical variable selection (Table 5) indicate that the proportion of population with access to an ITN in their household was the only indicator able to capture the effect of ITN interventions as it has the highest inclusion probability. This indicator was used to quantify the effect of ITNs on the parasitaemia odds change.

**Table 5:** Posterior inclusion probability for ITN coverage indicator for MIS 2014

Indicator	Probability of inclusion (%)
Proportion of households with at least one ITN	5.8
Proportion of households with at least one ITN for every two people	6.1
Proportion of population with access to an ITN in their household	42.7
Proportion of the population that slept under an ITN the previous night	4.7
Proportion of children under 5 years old who slept under an ITN the previous night	12.3
Proportion of existing ITNs used the previous night	0.2

*Abbreviations*: *MIS* Malaria Indicator Survey, *ITN* Insecticide Treated Net

### Effects of interventions on parasitaemia odds decline

The effects of interventions on the change of parasitaemia odds adjusted for socioeconomic status and changes in environmental conditions between the two surveys are showed in Table 6. Results demonstrate an important protective effect of interventions on the decrease of parasitaemia odds from 2009 to 2014. ITNs, IRS and ACTs were associated with a parasitaemia odds reduction of 19% (95% BCI: 18–29%), 78% (95% BCI: 67–84%), and 34% (95% BCI: 28–66%), respectively. Similarly, higher socio-economic status had a strong effect on parasitaemia odds reduction. More so, living in urban areas was associated with a decrease in malaria odds of 57% (95% BCI: 47–60%) compared to living in rural areas. On average, rainfall, day and night LST increased from 2009 to 2014, and these increases were significantly associated with increased parasitaemia odds. However, changes in the NDVI had no effect on changes in parasitaemia odds.

**Table 6:** Posterior estimates for the effect of interventions adjusted for socio-economic status and changes in climatic/environmental conditions

Covariate	OR (95% BCI)
Difference in LST (day)	1.10 (1.02–1.13)^a^
Difference in LST (night)	1.09 (1.03–1.18)^a^
Difference in NDVI	1.00 (0.94–1.08)
Difference in rainfall	1.14 (1.08–1.23)^a^
Area type (urban *vs* rural)	0.43 (0.40–0.53)^a^
Wealth index	0.54 (0.51–0.57)^a^
ITN	0.81 (0.71–0.82)^a^
IRS	0.22 (0.16–0.33)^a^
ACTs	0.66 (0.34–0.72)^a^
Spatial variance	0.63 (0.56–0.76)
Range (km)	35.4 (24.3–37.0)

*Abbreviations*: *ITNs* Insecticide Treated Nets, *ACTs* Artemisinin Combination Therapies, *IRS* Indoor Residual Spraying, *LST* Land Surface Temperature, *NDVI* Normalized Difference Vegetation Index

^a^Statistically important effect

**Fig. 5:**
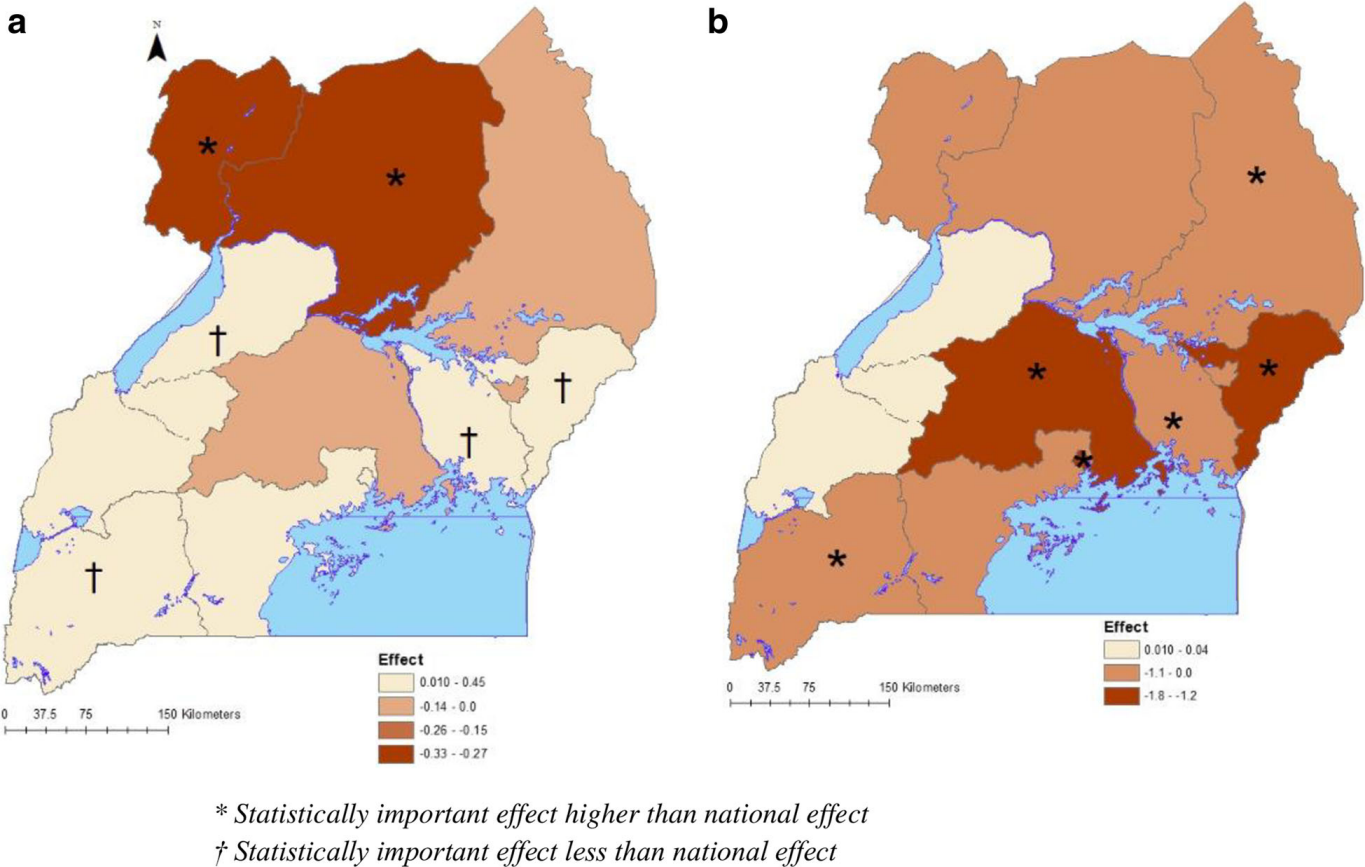
Spatially varying effects of interventions for ITNs (**a**) and ACTs (**b**)

Intervention effects on parasitaemia odds decline varied by region (Fig. 5). The effect of ITNs at regional level was significantly higher than the national effect in Mid-North and West Nile. ITNs’ effects were significantly lower in East-Central, Mid-Eastern, Mid-Western, and South-Western regions. Likewise, the effect of ACTs was significantly higher than the national average in most regions except in Central 1, Mid-North, Mid-Western, and West Nile regions.

## Discussion

In this study we have determined the spatio-temporal trends of parasitaemia odds and the effect of control interventions on the change of parasitaemia risk in Uganda during 2009–2014. Furthermore, we estimated the probability of parasitaemia risk decline and the number of infected children at the two survey time points.

Our study results showed a strong ITNs effect on parasitaemia risk reduction during 2009–2014 following a two-fold increase in coverage in the 5 years. These results support findings in similar malaria endemic settings [3]. This protective effect can be attributed to the physical barrier provided by ITNs to block mosquitoes from infecting humans with *Plasmodium* sporozoites, thus preventing parasites from completing their development cycle [19]. Also, the insecticide in ITNs reduces the lifespan of vectors when they come into contact, thus decreasing the chances of transmission [6]. Furthermore, the high coverage and utilization registered in the country may have achieved a ‘mass effect’ that reduces the mosquito population and thus protects people in communities who are not using ITNs but live in close proximity to households with ITNs [20, 21].

The high increase in ITNs coverage can be credited to increased donor support that funded ITNs purchase and distribution through effective distribution outreach channels [8]. These channels include mass distribution campaigns, antenatal care clinics, Expanded Program for Immunization (EPI), and commercial sale of subsidized ITNs through the private sector. These distribution channels have had an immediate success of raising the proportion of households possessing at least one ITN from less than 50% to more than 90%. In spite of the high ITN coverage across the country, ITN effects on parasitaemia odds reduction varied with region. Effects were highest in regions which were initially the most high burdened in 2009. The varying effects of interventions could be explained by regional heterogeneities in malaria transmission rates [22], ecology, and access to health services [23].

Furthermore, case management with ACTs was strongly associated with parasitaemia risk reduction following a three-fold increase in coverage during the study period. Prompt treatment of malaria with ACTs suppresses and kills malaria parasites in the body which prevents progression to severe disease, thus reducing transmission and subsequently parasitaemia load in the population [24]. In line with our study findings, Bhatt et al. [3] also found that ACTs together with ITNs were the most impactful interventions on malaria risk reduction in African endemic countries during 2000–2015. Also, effects of ACTs also varied with region. However, despite the two-fold increase in ACTs coverage in the 5 years, its coverage was still lower than targeted. This could possibly be attributed to supply chain constraints [25], the semi-regulated private health facilities and drug stores and the inadequate laboratory diagnostic capacity in most of the lower level facilities [8].

Indoor residual house spraying also had a very strong effect on parasitaemia odds reduction despite its coverage remaining static between 2009 and 2014. The endophilic behavior of the predominant *Anopheles* spp. mosquitoes makes this intervention highly effective in Uganda as vectors are killed by the insecticide as they rest on house walls after taking a blood meal [26]. The static coverage is perhaps explained by the high costs involved in IRS implementation. This prompted NMCP to roll out IRS gradually initially starting in 2009 with the 10 most high malaria burden districts located in the Mid-North region [8]. Following a significant reduction in malaria transmission in the 10 districts [27], IRS was later extended to another 14 high burden districts in the North-East, Mid-Eastern, and East-Central regions. Effectiveness of IRS on malaria risk reduction has been reported in other studies in Uganda [27], Kenya [28], Bioko, Equatorial Guinea and Mozambique [29].

Our results further showed that urban areas were associated with a decreased parasitaemia risk compared to rural areas. This could be explained by uneven access to healthcare services between urban and rural areas in developing countries [30]. In Uganda, lower level health facilities, which are the major source of health services in rural areas, are poorly equipped and understaffed [31]. On the other hand, urban areas are served by a much bigger network of better equipped higher level facilities both public and private. Indeed urbanization is one of the reasons that has been suggested as a strong possible causal factor of the downward trend of malaria risk in the pre-intervention period [32]. The effect of urbanization on socio-economic and landscape changes mitigates the risk of malaria transmission. The inverse relationship between urbanization and malaria risk has also been reported in other malaria endemic settings [32–35].

Higher socio-economic status was strongly associated with parasitaemia odds reduction. Related to this finding, our results also showed that the highest probability of parasitaemia decline was attained in Kampala region and the lowest in the North-East region. The former is the capital city and the most developed region, while the latter is the least developed and most hard-to-reach region in Uganda. Socio-economic status affects the ability to afford healthcare services, better housing conditions, and knowledge of malaria prevention [36], which are important determinants of severity and outcome of the disease. These results are in agreement with other studies that reported a higher burden of malaria among poor countries [37] and in hard-to-reach areas [6, 37]. This finding augments evidence that malaria is a disease associated with poverty [38, 39] and low socio-economic development [39–42].

Furthermore, increased land surface temperature and rainfall between 2009 and 2014 were associated with a higher parasitaemia risk. This result is expected since malaria is a vector-borne disease sensitive to changes in climatic conditions [2]. Temperature influences the speed of development of mosquitoes and *Plasmodium* parasites [43]. Rainfall is the most important driver of mosquito population dynamics and malaria transmission because it provides the optimal humidity and medium for mosquito fertilization and breeding [44, 45].

Although a reduction in parasitaemia risk was achieved in all regions, nevertheless, parasitaemia risk was still high in the regions of North-East, West Nile, and East-Central compared to other regions. This disproportionately high risk in these regions in spite of the high intervention coverage might be attributed to low socio-economic development [46], and limited access to health services [23]. In the case of East-Central region, rice growing practiced in this region has been documented as a potential driver of malaria risk transmission due to the large swamps that provide a favorable habitat for mosquito breeding [47]. Similarly, other studies have reported a higher malaria risk in settings with low socio-economic status [42], poor access to health services [39], and rice paddies [48].

The strong reduction in the estimated number of malaria-infected children may also underline the effect of increases in interventions coverage [9], urbanization [49], and generally improving socio-economic conditions [50].

## Conclusions

Our study demonstrates that malaria control interventions have had a strong effect on the decline of parasitaemia risk in Uganda during 2009–2014, albeit with varying magnitude in the regions. This success should be sustained by optimizing ITN coverage to achieve universal coverage and by timely replacing worn-out ITNs. NMCP should sustain the malaria prevention awareness campaigns through the use of Information, Education and Communication (IEC) materials to further promote the use of ITNs. In the high burden districts where IRS implementation is on-going, efforts should be made to ensure that all households are sprayed periodically every 6 months. NMCP should address the problems limiting ACTs coverage scale-up by providing free RDTs to all healthcare providers in line with the WHO ‘Test and Treat’ campaign, and increasing supervision for private health facilities. The varying intervention effects in different regions maybe an indication that interventions work differently in different regions of the country. This therefore calls for a better understanding of the environmental and entomological conditions in each region to tailor a combination of interventions suitable to local settings that will have maximum reduction on transmission. Also, in the regions where the risk remains disproportionately high, NMCP needs to conduct specific studies to understand human and/or vector behavior responsible for this problem. In these regions, other tools should be introduced such as chemoprevention especially in the high risk group of children less than 5 years and mass drug administration to reduce the parasite load in the population. In order to maximize intervention effects and avert reversal in malaria risk reduction, government and donor funded poverty reduction programs should prioritize regions/districts where socio-economic conditions are low. In summary, the ambitious targets of UMRSP 2014–2020 can be achieved if the country commits to implementing an integrated package to cover all aspects of disease prevention, management, and health. However, this will only be possible if the current funding portfolio is increased from the contemporary less than $1 average per head per year to the recommended $4 per head per year [51] equivalent to $140 million per year.

## Additional files

Additional file 1: Details of statistical models to estimate parasitaemia risk, effects of interventions on the change of parasitaemia risk, and spatially varying interventions effects. (DOCX 40 kb)

Additional file 2: Joint posterior distributions of the fitted statistical models. (DOCX 32 kb)

Additional file 3: Malaria intervention coverage in 2009 and 2014. Percentage of households with one ITN (a), percentage of households with at least 1 ITN for every two people (b), percentage of population with access to an ITN (c), percentage of population that slept under an ITN the previous night (d), percentage of children less than 5 years who slept under an ITN the previous night (e), proportion of fever episodes treated with any ACT (f). (PDF 211 kb)

## Abbreviations

ACTs: Artemisinin combination therapies
BCI: Bayesian credible intervals
DHS: Demographic health survey
EWES: Environmental monitoring system
GRUMP: Global rural-urban mapping project
IRS: Indoor residual spraying
ITNs: Insecticide treated nets
LST: Land surface temperature
MIS: Malaria indicator surveys
MODIS: Moderate resolution imaging spectroradiometer
MoH: Ministry of Health
NDVI: Normalized difference vegetation index
NMCP: National malaria control program
PMI: President’s malaria initiative
RS: Remote sensing
SSA: Sub-Saharan Africa
UMRSP: Uganda malaria reduction strategic plan (UMRSP)
WHO: World Health Organization
