# Supplementary material for: The contribution of malaria control interventions on spatio-temporal changes of parasitaemia risk in Uganda during 2009–2014
Source: Parasit Vectors. 2017 Sep 30;10:450. doi: 10.1186/s13071-017-2393-0 (PMC5622426; doi:10.1186/s13071-017-2393-0)
Supplement: Supplementary file 1 — Details of statistical models to estimate parasitaemia risk, effects of interventions on the change of parasitaemia risk, and spatially varying interventions effects. (DOCX 40 kb) [file 13071_2017_2393_MOESM1_ESM.docx]

**Statistical modeling details**

**A1. Estimating parasitaemia risk at two survey time periods**

A geostatistical model was developed to assess the effect of environmental/climatic factors on parasitaemia risk for the first survey. Let $Y_{1}(s_{i})$ be the number of children less than 5 years who tested positive in cluster $s_{i}$ in the first survey, and $N_{1}\left( s_{i} \right),$ the total number of children tested. We assume that $Y_{1}(s_{i})$ follows a Binomial distribution, that is, $Y_{1}\left( s_{i} \right)|N_{1}\left( s_{i} \right),\pi_{1}(s_{i})\sim Bin(N_{1}\left( s_{i} \right),\pi_{1}\left( s_{i} \right))$ $\forall$i ∈ 1,…,n_1_, where **s**={s_1_, s_2_,…,s_n_} is the set of locations surveyed, $s_{i}\subset R^{2}$ and $\pi_{1}(.)$ indicates the parasitaemia risk. A Bayesian geostatistical model to analyze parasitaemia risk was formulated as follows:

$\mathrm{logit}\left( \pi_{1}\left( s_{i} \right) \right)=\boldsymbol{\beta}_{\mathbf{1}}^{T}\mathbf{X}_{\mathbf{1}}\left( s_{i} \right)+\omega_{1}(s_{i})$, where $\mathbf{X}_{\mathbf{1}}\left( s_{i} \right)$ is the set of environmental/climatic predictors at location$s_{i}$, $\boldsymbol{\beta}_{\mathbf{1}}$ = ($\beta_{11}, \beta_{12},\ldots,\beta_{1k}$)^T^ is the vector of regression coefficients and ${\boldsymbol{\omega}_{1}=(\omega_{1}\left( s_{1} \right), \omega_{1}\left( s_{2} \right),\ldots, \omega_{1}(s_{n1}))}^{T}$ is a zero-mean latent spatial process that follows a multivariate normal distribution, that is, $\boldsymbol{\omega}_{1}\sim MVN(0,{\sigma_{1}^{2}R}_{1})$. R_1_ is the correlation matrix defined by an exponential parametric function of the distance $d_{\mathrm{ij}}$ between two location $s_{i}$ and$s_{j}$ that is, $R(s_{i}, s_{j})=exp(-d_{\mathrm{ij}}\rho_{1})$. The parameter $\sigma_{1}^{2}$ is the spatial variation and $\rho_{1}$ is a smoothing parameter that controls the rate of correlation decay with increasing distance. The range parameter was calculated by the ratio $\frac{3}{\rho_{1}}$ to estimate the minimum distance beyond which spatial correlation is negligible (<5%). Following standard formulation of Bayesian regression models, we assumed vague priors; an inverse-gamma for $\sigma_{1}^{2}$, a gamma prior distributions for $\rho_{1}$, and non-informative Gaussian distributions with mean 0 and variance 10^2^ for the regression coefficients. Thus, $\sigma_{1}^{2}$~IG(0.01,0.01), $\rho_{1}$~Gamma(2.01,1.01), $\beta_{1k}$~N(0, 10^2^), k=1,…,K..

To produce a smooth map, Bayesian kriging was employed to predict parasitaemia risk at unsampled locations on a 2x2 km^2^ grid using the predictive posterior distribution, p(**Y_0_**| **Y**) = $\int p\left( \mathbf{Y}_{\mathbf{0}} | \boldsymbol{\beta}_{\mathbf{1}}, \boldsymbol{\omega}_{0} \right)p\left( \boldsymbol{\omega}_{0} | \boldsymbol{\omega}_{1},\sigma_{1}^{2}, \rho_{1} \right)p\left( \boldsymbol{\beta}_{\mathbf{1}}\mathbf{,} \boldsymbol{\omega}_{1},\rho_{1}, \sigma_{1}^{2} | Y_{1}\left( s_{i} \right) \right)d\boldsymbol{\beta}_{\mathbf{1}}d\boldsymbol{\omega}_{0}d\boldsymbol{\omega}_{1}d\sigma_{1}^{2}d\rho_{1}$, where

**Y_0_**=($Y_{1}\left( s_{01} \right),Y_{1}\left( s_{02} \right),\ldots,Y_{1}\left( s_{0l} \right)$)^T^ is the number of infected children at unsampled location $\mathbf{s}_{\mathbf{0}}$ = {$s_{01}, s_{02}, \ldots,s_{0l}\}$ ,

$\boldsymbol{\omega}_{0}$ is the spatial random effect at $\mathbf{s}_{\mathbf{0}}$**.** The distribution of $\boldsymbol{\omega}_{0}$ given $\boldsymbol{\omega}_{1}$ is multivariate normal, that is, $p\left( \boldsymbol{\omega}_{0} | \boldsymbol{\omega}_{1},\sigma_{1}^{2}, \rho_{1} \right)$=MVN ($R_{01}R_{11}^{-1}U, {\sigma_{1}^{2}(R}_{01}-R_{01}R_{11}^{-1}R_{10})$), with $R_{11}$ = cor($\boldsymbol{\omega}_{1},\boldsymbol{\omega}_{1}$), $R_{01}$ = $R_{10}^{T}$ = cor($\boldsymbol{\omega}_{0},\boldsymbol{\omega}$) and p($Y\left( s_{0i} \right)|\boldsymbol{\beta}_{\mathbf{1}}\boldsymbol{,\omega}\left( s_{0i} \right)$)~$Bin(Y\left( s_{0i} \right),\pi_{0}\left( s_{0i} \right))$ , and thus logit ($\pi_{0}\left( s_{0i} \right))$=${\boldsymbol{\beta}_{\mathbf{1}}}^{T}\mathbf{X}\left( s_{0i} \right) \boldsymbol{+\omega}\left( s_{0i} \right)$.

For mapping purposes, predictions were made for 52,794 pixels covering a regular grid of Uganda.

Using a geostatistical model similar to the one described above, estimates of malaria risk were obtained for the second survey. Similarly, a Binomial distribution was assumed for the number of positive children$Y_{2}(s_{i}^{'})$, that is, $Y_{2}\left( s_{i}^{'} \right)|N_{2}\left( s_{i}^{'} \right),\pi_{2}\left( s_{i}^{'} \right)\sim Bin\left( N_{2}\left( s_{i}^{'} \right),\pi_{2}\left( s_{i}^{'} \right) \right),$ $\forall i \in1,\ldots,n_{2}$ where $\mathbf{s}^{\mathbf{'}}=\{s_{1}^{'}, s_{2}^{'},\ldots,s_{n_{2}}^{'}\}$ is the set of locations sampled in the second survey, which is different from$\mathbf{s}$. $\pi_{2}(s_{i}^{'})$ was modeled as a function of the environmental factors and a spatial process $\boldsymbol{\omega}_{\mathbf{2}}$, that is, $\boldsymbol{\omega}_{\mathbf{2}}\sim MVN(0,{\sigma_{2}^{2}R}_{2})$ with spatial variance $\sigma_{2}^{2}$ and scaling parameter $\rho_{2}$. On the logit scale, this takes the form, $\mathrm{logit}\left( \pi_{2}\left( s_{i}^{'} \right) \right)= \boldsymbol{\beta}_{\mathbf{2}}^{T}\mathbf{X}_{\mathbf{2}}\left( s_{i}^{'} \right)+\omega_{2}(s_{i}^{'})$. Also, prediction of parasitaemia risk for the second survey was carried out using the 2x2 km^2^ resolution grid described above.

**A2. Modeling the effects of interventions on the change of parasitaemia risk**

The change of parasitaemia risk was modeled on the logit scale as a function of the difference in climatic conditions between the two survey times, the effect of intervention coverage, the socio-economic status and area type in the second survey, that is; $logit(\pi_{2}\left( s_{i}^{'} \right)=Z({s'}_{i}) +{\boldsymbol{\beta(X}_{\mathbf{2}}\left( s_{i}^{'} \right)-\mathbf{X}_{\mathbf{1}}\left( s_{i}^{'} \right))}^{T}+\alpha_{1}\mathrm{ITN}\left( s_{i}^{'} \right)+\alpha_{2}\mathrm{IRS}\left( s_{i}^{'} \right)+\alpha_{3}ACT(s_{i}^{'})+\gamma_{1}\mathrm{Area}\left( s_{i}^{'} \right)+\gamma_{2}\mathrm{wealth}\left( s_{i}^{'} \right)+\omega_{c}(s_{i}^{'}))$,

where Z(${s'}_{i}$) = logit($\pi_{1}({s_{i}}^{'})$), $\mathrm{ITN}\left( s_{i}^{'} \right)$ is the coverage indicator identified through a variable selection procedure among six ITN use and ITN ownership indicators, $\mathrm{IRS}\left( s_{i}^{'} \right)$ represents the proportion of sprayed households in cluster $s_{i}^{'}$, $ACT(s_{i}^{'})$ is the proportion of fevers treated with any ACT, and $\boldsymbol{\omega}_{\mathbf{c}}(s_{i}^{'})$ corresponds to the latent spatial process, that is, $\boldsymbol{\omega}_{\mathbf{c}}\sim MVN(0,{\sigma_{c}^{2}R}_{c})$ with spatial variance $\sigma_{c}^{2}$. The coefficients $\alpha_{1}$, $\alpha_{2}$ and $\alpha_{3}$ measure the effect of interventions on the change in parasitaemia risk, thus, $exp(\alpha_{1})$, $exp(\alpha_{2})$ and $exp(\alpha_{3})$ are the expected change in odds of parasitaemia (second survey versus first survey) associated with a 1% increase in the coverage of ITNs, IRS and ACT, respectively. $\mathrm{Area}\left( s_{i}^{'} \right)$ is a binary variable indicating whether $s_{i}^{'}$ is an urban or rural cluster, and $\mathrm{wealth}\left( s_{i}^{'} \right)$ is the median wealth score of cluster $s_{i}^{'}$. Coefficients $\gamma_{1}\mathrm{and}\gamma_{2}$ are covariate effects quantifying the effect of $\mathrm{Area}\left( s_{i}^{'} \right)$ and $\mathrm{wealth}\left( s_{i}^{'} \right)$ on the parasitaemia odds reduction. $\boldsymbol{\omega}_{\mathbf{c}}$**(**$\mathbf{s'}$**)** are spatial random effects modeled by a Gaussian process as $\boldsymbol{\omega}_{\mathbf{c}}$**~**MVN(0,$\sigma_{c}^{2}R_{c}$)

We assume an inverse gamma prior distribution for $\sigma_{c}^{2},$ a gamma distribution for the parameter $\rho_{c}$, and normal priors for the regression coefficients $\boldsymbol{\beta}$**,** $\alpha_{1}$, $\alpha_{2}$, $\alpha_{3}$, $\gamma_{1},\gamma_{2}$.

Parasitaemia risk during the first survey $\pi_{1}(.)$ was not directly available at locations $\mathbf{s}^{\mathbf{'}}$ of the second survey. We addressed this spatial misalignment problem by predicting parasitaemia risk during the first period at the locations of the second survey using the Bayesian kriging. The estimation error of parasitaemia prediction was taken into account in the modeling as a measurement error in the covariate.

The joint posterior distribution of the parameters was obtained by

p($\boldsymbol{\beta,}\boldsymbol{\beta}_{1}, Z\left( s^{'} \right),\alpha_{1}$,$\alpha_{2}$,$\alpha_{3}$,$\gamma_{1},\gamma_{2}, \boldsymbol{\omega}_{1}\left( s \right), \boldsymbol{\omega}_{\mathbf{1}}, \omega_{c},\sigma_{c}^{2},\rho_{c}, \sigma_{1}^{2},\rho_{1}\left| Y_{2}\left( s^{'} \right) \right)\propto p\left( Y_{2}\left( s^{'} \right) | Z\left( s^{'} \right)\boldsymbol{,\beta},\alpha_{1},\alpha_{2},\alpha_{3},\gamma_{1},\gamma_{2},\omega_{c} \right)p\left( Z\left( s^{'} \right) | \boldsymbol{\beta}_{1},\boldsymbol{\omega}_{1} \right)p\left( \boldsymbol{\omega}_{1} | \boldsymbol{\omega}_{1} \right)p\left( \boldsymbol{\omega}_{1} | \sigma_{1}^{2},\rho_{1} \right)p\left( \boldsymbol{\omega}_{\mathbf{c}} | \sigma_{c}^{2},\rho_{c} \right)p\left( \boldsymbol{\beta} \right)p\left( \boldsymbol{\beta}_{1} \right)$

$p\left( \alpha_{1} \right)p\left( \alpha_{2} \right)p\left( \alpha_{3} \right)p\left( \gamma_{1} \right)p(\gamma_{2})p\left( \sigma_{1}^{2} \right)p(\rho_{1})$p($\sigma_{c}^{2}$)p($\rho_{c}$)

**A3. Spatially varying interventions effects**

In order to estimate the intervention effects at regional level and account for potential interactions with endemicity levels, a second model was fitted in which we estimated intervention effects at regional level. The model was expressed as;

$\mathrm{logit}\left( \pi_{2}\left( s_{i}^{'} \right) \right)=Z\left( s_{i}^{'} \right)+\boldsymbol{\beta}\left( \mathbf{X}_{\mathbf{2}}\left( s_{i}^{'} \right)-\mathbf{X}_{\mathbf{1}}\left( s_{i}^{'} \right) \right)+\alpha_{1}\left( A_{s_{i}^{'}} \right)\mathrm{ITN}\left( s_{i}^{'} \right)+\alpha_{2}(A_{s_{i}^{'}})IRS\left( s_{i}^{'} \right)+\alpha_{3}\left( A_{s_{i}^{'}} \right)\mathrm{ACT}\left( s_{i}^{'} \right)+\omega_{c}(s_{i}^{'})$.

The effects of interventions are defined at regional level and denoted as $\alpha_{k}\left( A_{s_{i}^{'}} \right), (k=1,2,3)$ where $A_{s_{i}^{'}}$ is the region where $s_{i}^{'}$ falls. Each $\alpha_{k}(A_{i})$ was written as the sum of a conditional autoregressive effect that takes into account the similarity of the effects across the regions and an independent random component, that is, $\alpha_{k}\left( A_{i} \right)= \alpha_{k}^{c}\left( A_{i} \right)+\varepsilon_{k}(A_{i})$, where $p(\alpha_{k}^{c}\left( A_{i} \right)|\alpha_{k}^{c}\left( A_{j} \right), i\neq j, \tau_{\mathrm{kc}})\equiv N(\frac{1}{n_{i}}\sum_{i\sim j} \alpha_{k}^{c}\left( A_{j} \right),\frac{\sigma_{\mathrm{kc}}^{2}}{n_{i}})$ with $i\sim j$ indicates the $A_{j}$ areas neighboring $A_{i}$ , and $\varepsilon_{k}\left( A_{i} \right)\sim N(0,\sigma_{\varepsilon}^{2})$.

**A4. Bayesian variable selection**

To choose the most important ITN coverage indicator and functional form that explains the maximum variation in parasitaemia odds change, Bayesian variable selection using stochastic search was implemented. For each ITN coverage covariate$X_{p}$, a categorical indicator parameter $I_{p}$ was introduced to represent exclusion of the variable from the model$\left( I_{p}=0 \right)$, inclusion in linear $\left( I_{p}=1 \right)$ or categorical $\left( I_{p}=2 \right)$ forms. $I_{p}$ has a probability mass function $\prod_{j=0}^{2} \pi_{j}^{\delta_{j}\left( I_{p} \right)}$, where $\pi_{j}$ denotes the inclusion probabilities of functional form j (j=0,1,2) so that $\sum_{j=0}^{2} \pi_{j}=1$ and $\delta_{j}\left( . \right)$ is the Dirac function, $\delta_{j}\left( I_{p} \right)=\left\{ \begin{aligned} 1, &if I_{p}=j \\ 0, &if I_{p} \neq j \end{aligned} \right. .$A spike and slab prior distribution was assumed for the regression coefficients. In particular for the coefficient $\beta_{p}$ of the corresponding variable X_p_ in linear form, we assumed $\beta_{p}\sim\delta_{1}\left( I_{p} \right)N\left( 0,\tau_{p}^{2} \right)+\left( 1-\delta_{1}\left( I_{p} \right) \right)N\left( 0,\vartheta_{0}\tau_{p}^{2} \right)$ that is a non-informative prior for $\beta_{p}$ if $X_{p}$ is included in the model in linear form (slab) and an informative normal prior shrinking $\beta_{p}$ to zero (spike) if $X_{p}$ is excluded from the model, setting $\vartheta_{0}$to be a large number, e.g, 10^5^. Likewise, for the coefficients {$\left. \beta_{p,l} \right\}_{l=1,..,L}$ corresponding to the categorical form of X_p_ with L categories,$\beta_{p,l}\sim\delta_{2}\left( I_{p} \right)N\left( 0,\tau_{p,l}^{2} \right)+\left( 1-\delta_{2} \right)N(0,\vartheta_{0}\tau_{p,l}^{2})$ was assumed. For inclusion probabilities, a non-informative Dirichlet distribution was adopted with hyper parameter$\alpha={(1,1,1)}^{T}$, that is,$\boldsymbol{\pi}={(\pi_{0},\pi_{1},\pi_{2})}^{T}\sim Dirichlet\left( 3,\alpha\right).$ We also assumed inverse Gamma priors for the precision hyper parameters $\tau_{p}^{2}$ and $\tau_{p,l}^{2}$, $l=1,\ldots,L$.
