# Supplementary material for: The contribution of malaria control interventions on spatio-temporal changes of parasitaemia risk in Uganda during 2009–2014
Source: Parasit Vectors. 2017 Sep 30;10:450. doi: 10.1186/s13071-017-2393-0 (PMC5622426; doi:10.1186/s13071-017-2393-0)
Supplement: Supplementary file 2 — Joint posterior distributions of the fitted statistical models. (DOCX 32 kb) [file 13071_2017_2393_MOESM2_ESM.docx]

**Joint posterior distributions**

**A1. Estimating parasitaemia risk at first survey**

p($\boldsymbol{\beta}_{\mathbf{1}}$**,**$\boldsymbol{\omega}_{\mathbf{1}}, \rho_{1}$,$\sigma_{1}^{2}|\mathbf{Y}_{\mathbf{1}}$) ∝L($\boldsymbol{\beta}_{\mathbf{1}}$,$\boldsymbol{\omega}_{\mathbf{1}}, \rho_{1}$, $\sigma_{1}^{2};\mathbf{Y}_{\mathbf{1}}$) p($\boldsymbol{\beta}_{\mathbf{1}}$) p$\boldsymbol{(\omega}_{1}|\sigma_{1}^{2}, \rho_{1}$) p($\sigma_{1}^{2}$) p($\rho_{1}$), where L($\boldsymbol{\beta}_{\mathbf{1}}$,$\boldsymbol{\omega}_{\mathbf{1}}, \rho_{1}$, $\sigma_{1}^{2};\mathbf{Y}_{\mathbf{1}}$) is the likelihood, p($\boldsymbol{\beta}_{\mathbf{1}}$), p$(\boldsymbol{\omega}_{1}|\sigma_{1}^{2}, \rho_{1}$), p($\sigma_{1}^{2}$) and p($\rho_{1}$) are prior distributions of regression parameters, spatial random effects, variance and correlation parameters, respectively.

p($\boldsymbol{\beta}_{\mathbf{1}}$**,**$\boldsymbol{\omega}_{1}, \rho_{1}$,$\sigma_{1}^{2}|\mathbf{Y}_{\mathbf{1}}$ ) ∝ $\prod_{i=1}^{n1}{\pi_{1}\left( s_{i} \right)}^{\mathbf{Y}_{\mathbf{1}}}({1-\pi_{1}\left( s_{i} \right)}^{n1-\mathbf{Y}_{\mathbf{1}}})$det($R_{1}$)^-1^exp(-$\frac{1}{2}{\rho_{1}}^{T}{R_{1}^{-1}\rho}_{1}$)($\sigma_{1}^{2}$)^-(a+1)^exp($-\frac{b}{\sigma_{1}^{2}}$), where $\pi_{1}\left( s_{i} \right)= \frac{exp(\boldsymbol{\beta}_{\mathbf{1}}^{T}\mathbf{X}_{\mathbf{1}}\left( s_{i} \right)+\omega_{1}(s_{i}))}{1+exp(\boldsymbol{\beta}_{\mathbf{1}}^{T}\mathbf{X}_{\mathbf{1}}\left( s_{i} \right)+\omega_{1}(s_{i}))}$

**A1. Estimating parasitaemia risk at second survey**

p($\boldsymbol{\beta}_{\mathbf{2}}$**,**$\boldsymbol{\omega}_{2}, \rho_{2}$,$\sigma_{2}^{2}|\mathbf{Y}_{\mathbf{2}}$) ∝L($\boldsymbol{\beta}_{\mathbf{2}}$,$\boldsymbol{\omega}_{2}, \rho_{2}$, $\sigma_{2}^{2};\mathbf{Y}_{\mathbf{2}}$) p($\boldsymbol{\beta}_{\mathbf{2}}$) p$\boldsymbol{(\omega}_{2}|\sigma_{2}^{2}, \rho_{2}$) p($\sigma_{2}^{2}$) p($\rho_{2}$), where L($\boldsymbol{\beta}_{\mathbf{2}}$,$\rho_{2}$, $\sigma_{2}^{2};\mathbf{Y}_{\mathbf{2}}$) is the likelihood, and p($\boldsymbol{\beta}_{\mathbf{2}}$), p$(\boldsymbol{\omega}_{2}|\sigma_{2}^{2}, \rho_{2}$), p($\sigma_{2}^{2}$) and p($\rho_{2}$) are the prior distributions of regression parameters, spatial random effects, variance and correlation parameters, respectively.

p($\boldsymbol{\beta}_{\mathbf{2}}$**,**$\boldsymbol{\omega}_{2}, \rho_{2}$,$\sigma_{2}^{2}|\mathbf{Y}_{\mathbf{2}}$ ) ∝ $\prod_{i=1}^{n2}{\pi_{2}\left( s_{i} \right)}^{\mathbf{Y}_{\mathbf{2}}}({1-\pi_{2}\left( s_{i} \right)}^{n2-\mathbf{Y}_{\mathbf{2}}})$det($R_{2}$)^-1^exp(-$\frac{1}{2}{\rho_{2}}^{T}{R_{2}^{-1}\rho}_{2}$)($\sigma_{2}^{2}$)^-(a+1)^exp($-\frac{b}{\sigma_{2}^{2}}$), where $\pi_{2}\left( s_{i} \right)= \frac{exp(\boldsymbol{\beta}_{\mathbf{2}}^{T}\mathbf{X}_{\mathbf{1}}\left( s_{i} \right)+\omega_{2}(s_{i}))}{1+exp(\boldsymbol{\beta}_{\mathbf{2}}^{T}\mathbf{X}_{\mathbf{2}}\left( s_{i} \right)+\omega_{2}(s_{i}))}$

**A2. Modeling the effects of interventions on the change of parasitaemia risk**

p($\boldsymbol{\beta,}\boldsymbol{\beta}_{1}, Z\left( s^{'} \right),\alpha_{1}$,$\alpha_{2}$,$\alpha_{3}$,$\gamma_{1},\gamma_{2}, \omega_{c}\left( s^{'} \right), \boldsymbol{\omega}_{1}\left( s \right), \boldsymbol{\omega}_{\mathbf{1}}\left( s^{'} \right), \boldsymbol{\omega}_{\mathbf{c}},\sigma_{c}^{2},\rho_{c}, \sigma_{1}^{2},\rho_{1}\left| Y_{2}\left( s^{'} \right) \right)\propto p\left( Y_{2}\left( s^{'} \right) | \boldsymbol{\beta},\alpha_{1},\alpha_{2},\alpha_{3},\gamma_{1},\gamma_{2},Z\left( s^{'} \right),\omega_{c}\left( s^{'} \right) \right)p\left( Z\left( s^{'} \right) | \boldsymbol{\beta}_{1},\boldsymbol{\omega}_{1}\left( s^{'} \right) \right)p\left( \boldsymbol{\omega}_{1}\left( s^{'} \right) | \boldsymbol{\omega}_{1}\left( s \right) \right)p\left( \boldsymbol{\omega}_{1}\left( s \right) | \sigma_{1}^{2},\rho_{1} \right)$

$p\left( \omega_{c}\left( s^{'} \right) | \sigma_{c}^{2},\rho_{c} \right)p\left( \boldsymbol{\beta} \right)p\left( \boldsymbol{\beta}_{1} \right)p\left( \alpha_{1} \right)p\left( \alpha_{2} \right)p\left( \alpha_{3} \right)p\left( \gamma_{1} \right)p(\gamma_{2})p\left( \sigma_{1}^{2} \right)p(\rho_{1})$p($\sigma_{c}^{2}$)p($\rho_{c}$)

**A3. Spatially varying interventions effects**

p($\boldsymbol{\beta,}\boldsymbol{\beta}_{1}, Z\left( s^{'} \right),\boldsymbol{\alpha}_{\mathbf{1}}\left( A_{s^{'}} \right)$, $\boldsymbol{\alpha}_{\mathbf{2}}\left( A_{s^{'}} \right)$, $\boldsymbol{\alpha}_{\mathbf{3}}\left( A_{s^{'}} \right)$,$\boldsymbol{\omega}_{\mathbf{c}}\left( s^{'} \right), \boldsymbol{\omega}_{1}\left( s \right), \boldsymbol{\omega}_{\mathbf{1}}\left( s^{'} \right), \boldsymbol{\omega}_{\mathbf{c}},\sigma_{k_{c}}^{2},\rho_{c}, \sigma_{1}^{2},\rho_{1}\left| Y_{2}\left( s^{'} \right) \right)\propto p\left( Y_{2}\left( s^{'} \right) | \boldsymbol{\beta},\boldsymbol{\alpha}_{\mathbf{1}}\left( A_{s^{'}} \right), \boldsymbol{\alpha}_{\mathbf{2}}\left( A_{s^{'}} \right), \boldsymbol{\alpha}_{\mathbf{3}}\left( A_{s^{'}} \right),Z\left( s^{'} \right),\boldsymbol{\omega}_{\mathbf{c}}\left( s^{'} \right) \right)p\left( Z\left( s^{'} \right) | \boldsymbol{\beta}_{1},\boldsymbol{\omega}_{1}\left( s^{'} \right) \right)p\left( \boldsymbol{\omega}_{1}\left( s^{'} \right) | \boldsymbol{\omega}_{1}\left( s \right) \right)$

$p\left( \boldsymbol{\omega}_{1}\left( s \right) | \sigma_{1}^{2},\rho_{1} \right)p\left( \boldsymbol{\omega}_{\mathbf{c}}\left( s^{'} \right) | \sigma_{k_{c}}^{2},\rho_{c} \right)p\left( \boldsymbol{\beta} \right)p\left( \boldsymbol{\beta}_{1} \right)p\left( \boldsymbol{\alpha}_{\mathbf{1}}\left( A_{s^{'}} \right) \right)p\left( \boldsymbol{\alpha}_{\mathbf{2}}\left( A_{s^{'}} \right) \right)p\left( \boldsymbol{\alpha}_{\mathbf{3}}\left( A_{s^{'}} \right) \right)p\left( \sigma_{1}^{2} \right)p(\rho_{1})$p($\sigma_{k_{c}}^{2}$)p($\rho_{c}$)

Prior distributions for model parameters were assumed as in A2 above except for the spatially varying interventions effects $\alpha_{k}\left( A_{s^{'}} \right)$ for which a CAR prior distribution was adopted, implying that each $\alpha_{k}\left( A_{i} \right)$ conditional on $\alpha_{k}\left( A_{j} \right)$ follows a normal distribution with mean equal to the average of neighboring regions $A_{j}$ and variance inversely proportional to the number of neighbor regions n_i_, that is $p\left( \alpha_{k}\left( A_{i} \right) | \alpha_{k}\left( A_{j} \right), i\neq j, \tau_{\mathrm{kc}} \right)\sim N\left( \frac{1}{n_{i}}\sum_{i\sim j} \alpha_{k}\left( A_{j} \right),\frac{\sigma_{\mathrm{kc}}^{2}}{n_{i}} \right).$
