# Supplementary material for: The contribution of malaria control interventions on spatio-temporal changes of parasitaemia risk in Uganda during 2009–2014
Source: Parasit Vectors. 2017 Sep 30;10:450. doi: 10.1186/s13071-017-2393-0 (PMC5622426; doi:10.1186/s13071-017-2393-0)
Supplement: Supplementary file 3 — Malaria intervention coverage in 2009 and 2014. Percentage of households with one ITN (a), percentage of households with at least 1 ITN for every two people (b), percentage of population with access to an ITN (c), percentage of population that slept under an ITN the previous night (d), percentage of children less than 5 years who slept under an ITN the previous night (e), proportion of fever episodes treated with any ACT (f). (PDF 211 kb) [file 13071_2017_2393_MOESM3_ESM.pdf]

Malaria intervention coverage in 2009 (top) and 2014 (bottom)

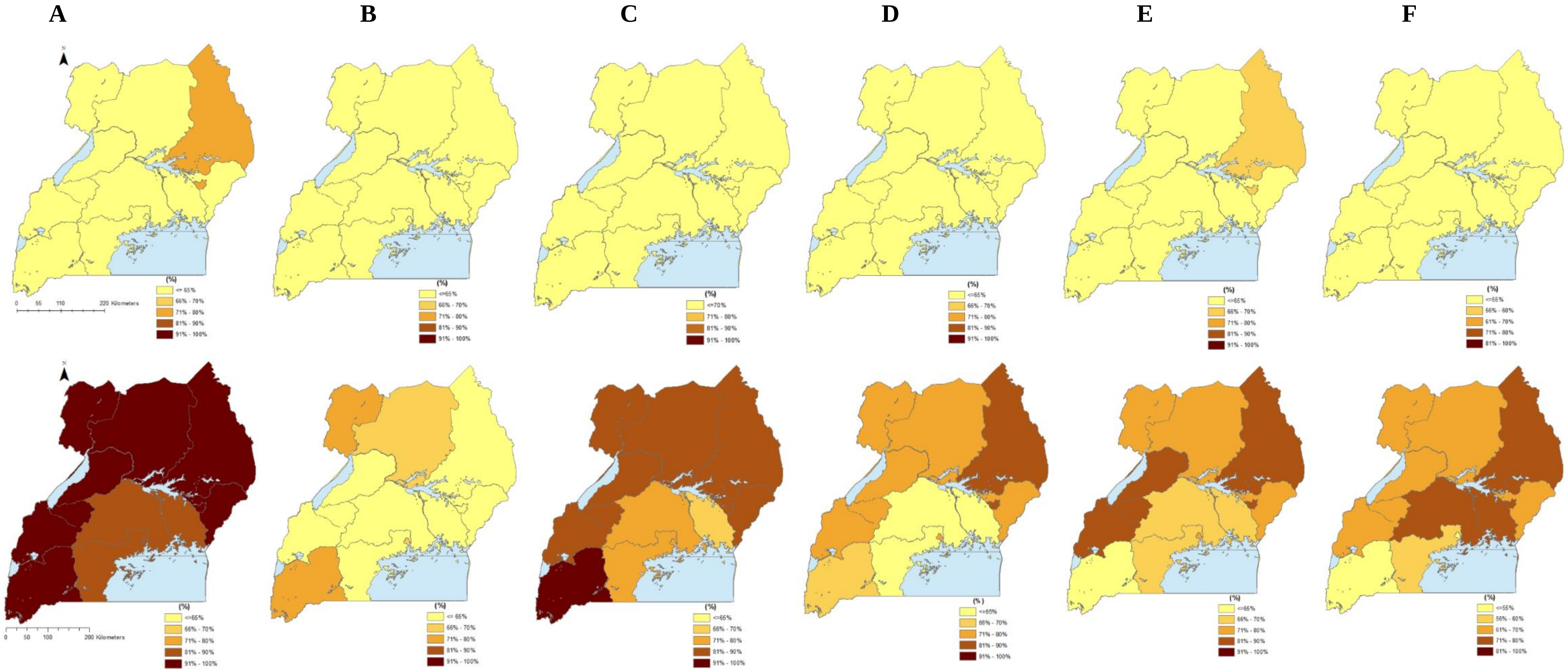

Percentage of households with one ITN (A), percentage of households with at least 1 ITN for every two people (B), percentage of population with access to an ITN (C), percentage of population that slept under an ITN the previous night (D), percentage of children less than 5 years who slept under an ITN the previous night (E), proportion of fever episodes treated with any ACT (F)
